# Comprehensive View of the Human Mating Process Among Young Couples in Isfahan-Iran: An Explanatory Mixed-Method Study

**DOI:** 10.5812/ircmj.10445

**Published:** 2013-12-05

**Authors:** Effat Merghati Khoei, Tayebe Ziaei, Mehrdad Salehi, Ziba Farajzadegan

**Affiliations:** 1 School of Public Health, Tehran University of Medical Sciences, Tehran, IR Iran; 2 Nursing and Midwifery College, Isfahan University of Medical Sciences, Isfahan, IR Iran; 3 Psychiatry Department, Isfahan University of Medical Sciences, Isfahan, IR Iran; 4 Community Medicine Department, Isfahan University of Medical Sciences, Isfahan, IR Iran

**Keywords:** Humans, Mating, Young Adult, Couples, Iran

## Abstract

**Background:**

Heterosexual relationship is the main component of mate selection. Regardless of the importance of mate favorites, little is known about exact valued criteria in potential mates.

**Objectives:**

This study was designed to comprehensively explain the theoretical view of the human mating process.

**Materials and Methods:**

This was as an explanatory mixed–method study. The first phase was a cross-sectional quantitative study with two Farsi-modified versions of instruments: preferences concerning potential mates and factors of choosing a mate; content analysis was the second phase. The quantitative phase of this study consisted of 202 dating couples, decided to get married. The qualitative phase consisted of 28 participants who acquired the extreme scores (highest and lowest) in the first phase.

**Results:**

Average age of marriage for women and men was 23.04 and 26.41 respectively; the actual age difference was 3.37 years (women younger than men). The results of this study in support of evolution-based theory explained that, age is a preference and choosing an older husband and a younger wife is due to having reproductive capacity. Also, they mentioned that appearance is necessary for men because of sexual attraction, not as a prediction for the next generation appearance. In both phases of this study, both genders had a strong emphasis on “chastity” in a potential mate. Results showed that, men preferred a mate who was a good housewife, capable of cooking, and women preferred a mate with “Good earning capacity”, “Good financial prospect” “university education”, “Favorable social status” and “Industriousness”.

**Conclusions:**

The results confirmed that for a comprehensive view in human mating process, we need a combined theoretical approach as well as qualitative and quantitative study to explore the real meaning of each preference in a mate.

## 1. Background

Marriage is a custom in which mate selection occurs. Therefore, a socially-acceptable sexual partner must be defined (1). Mate selection is a process affected by a variety of factors such as socio-economic, socio-cultural and individual traits. Mate selection preferences vary from one society to another which makes it complex (1, 2). Partner selection prototypes have always been the center of interest among researchers. Sociologists focus on the effects of educational institutions on the mate choice as well as distribution of the wealth. Evolutionary biologists believe that mate selection is a nonrandom and directional process. Geneticists point out the distribution of genotypes within and between families (3-6).

A number of theories describe the strategies of mate selection. One of them believed that people unconsciously look for qualities in mates that would be similar to images or prototypes of their opposite-sex parent; others proposed that people try to choose a spouse who can complete the characteristics they lack; another group point out that people seek for similarities in mate selection. Exchange and equity theories noted that people like the partners who exchange valuable resources (7, 8). Two main theoretical approaches are evolutionary and social structures. While the evolutionary approach explains the variety of traits through evolution, the social structural approach describes it in cultural exchanges and genders’ different believes. A combined theoretical approach offers a comprehensive view of the human mating process (9, 10).

Gender differences have been one of the mate preferences for several decades. Men tend to value physical attractiveness and women tend to value social status; however, depending on how someone asks them about their preferences, their answer might be different (11-14). Time and culture can affect these preferences due to the changing position and empowerment of women, broad access to TV, cinema and mass media, widespread usage of birth control which can be responsible for changing the nature of the families and workplaces. For example,” college graduate” was highly selected as a criterion, since it can be a guarantee of income. “Good earning capacity”, “good financial prospect” and “dependable character” were highly chosen as well. Conversely, the values of “chastity or no previous sexual intercourse” and “being a good housewife” were diminished (15-17). Iran is a multi-subcultural country with socio-cultural and educational changes in the recent years (18). Nevertheless, the family role in Iranian mate selection pattern is bold and arranged marriage is dominant; young people have dominant roles in “seeking the mate”, but in the final decision called “mate selection” their parents are dominant and we can see their footsteps (19).

## 2. Objectives

Researches in the field of mating preferences among Iranian young people have shown similarities and differences in mate preferences with other countries, because of different data collection methods. On the other hand, it is hard to compare the similarities and differences of Iranian people as an Islamic and multi-subcultural country with other people in the world. Therefore, a mixed-method study was designed to explain the appropriate theoretical approach that leads to a comprehensive view of the human mating process. The quantitative phase was designed to identify the mate selection criteria in dating couples (men and women who decided to get married) and qualitative phase was designed to explain the reasons of mate preferences that the quantitative instruments could not evaluate, more completely. Finally, the mixed results tried to find the appropriate theoretical approach that leads to a comprehensive view of the human mating process.

## 3. Materials and Methods

This explanatory mixed-method study was conducted based on a permit issued by the ethical-scientific committee of Isfahan, Iran, in 2012 (registered number: 389465). The first phase was a cross-sectional quantitative study, and qualitative (content analysis) was the second. The second phase allowed the participants to freely express their opinions about the mate selection preferences.

The quantitative phase of this study consisted of 202 dating couples who decided to get married who had registered in Yas, a premarital laboratory and consulting center in Isfahan, Iran. This center provides two services before marriage; marital laboratory tests, and premarital education and counseling courses. The inclusion criteria were: age between 18 - 35 years, education level of diploma and more, proceeding for the first marriage, volunteering to participate in this research (n = 212 couples). Exclusion criteria were: psychological disorder or diseases (two men and two women), and having bad events during the past 6 months on their report (one woman). Therefore, these samples and their spouses were excluded (n = 5 couples). Qualitative participant were selected from the quantitative phase. It means that, based on the score of the questionnaire, we chose 28 participants who took the extreme scores (highest and lowest).

The couples were asked to read, sign and print their names on an informed consent sheet. All the information was kept confidential according to the ethical committee protocol. Farsi versions of two questionnaires were used in the quantitative phase: 1- “factors in choosing a mate”, consisting of three parts (biographical data, information about the preferred age and age difference of the couple, and 23 characteristics participants should rate). A four-point scale was used (indispensable: 3 points; important, but not indispensable: 2 points; desirable, but not very important: 1 point; irrelevant or unimportant: 0 points). 2- “Preferences concerning potential mates” including 13 characteristics in a mate that participants were requested to rank them from 1 to 13. They had to give “1” to the most desirable characteristic in a potential mate (not necessarily the present mate) and “13” the least desirable one. Validity and reliability of the Farsi version was assessed in an unpublished Ph.D. dissertation by authors. Cronbach’s alpha coefficient for these 23 items was 74. Deep individual interview and focus group discussion were used in the qualitative phase.

To analyses the quantitative data, spss version16 was used. Wilcoxon test was used to compare wives and husbands. Effect size index (Cohen’s (1988), with |0.20| = small, |0.50| = medium, |0.80| = large) described the observed effects (20-22). In all interpretations, statistical significance was P < 0.05.

d = effect size calculated via the following formula

** Effect size index = d = x1 - x2/mean ± SD

Mean ± SD = sd1 + sd2/2

Content analysis was used in the qualitative phase of this study. This is a qualitative technique for a subjective explanation of content of a text data via a process of coding and identifying the themes. In the qualitative analysis, the recorded interviews were transcribed word by word, and then moved from the condensed meaning unit, coding, subcategorizing, categorization and themes (23).

## 4. Results

### 4.1. Quantitative Phase

Table 1 shows that there were gender differences in the couples’ ages, wives being younger than their husbands (23.04 ± 2.82 vs. 26.41 ± 3.1), which had large effect sizes (1.13). There were gender differences in the marriage age preference for girls and boys. Women preferred younger age of marriage for girls and boys than men; but there was no gender difference in the age difference preferences; wives preferred their husbands to be older (4.01 years) and husbands also preferred to be older themselves (3.88 years). In Tables 2, 3 and 4 we see the gender differences of mate preferences ranking in some criteria. 

**Table 1.** Ages and age Preferences for Marriage ^a ^

	Mean (SD)	t	P Value	D ^b^
**Age of couples**		17.62	< 0.0001	1.13
Women	23.04 (2.82)			
Men	26.41 (3.1)			
**Age preference for girls’ marriage**		8.12	< 0.0001	0.60
Women	23.16 (2.06)			
Men	24.58 (2.63)			
**Age preference for boys’ marriage**		5.65	< 0.0001	0.46
Women	24.27 (2.78)			
Men	25.25 (2.53)			
**Preference of age difference between wife and husband**		1.34	(0.18) NS ^c^	0.12
Women	4.01 (1.6)			
Men	3.88(1.68)			
**Marriage Age of couples**		17.61	< 0.0001	1.12
Women	23.01 (2.81)			
Men	26.38 (3.1)			

^a^ Means for all ages are expressed in years.

^b^ d = Cohen’s (1988) effect size index.

^c^ Abbreviation: NS, not significant.

**Table 2.** Gender Differences in Mate Preferences: Ranking Instrument

Mate Preference	Mean (SD)	Wilcoxon’s Gender Differences		
		z	P Value	d
**Kindness and sympathy**	-	0.71 ^b^	0.47 (NS)	.04
wife	2.19 (1.58)	-	-	-
husband	2.12 (1.64)	-	-	-
**Religiousness**	-	1.77 ^a^	0.07 (NS)	.12
wife	5.11 (4.03)	-	-	-
husband	5.63 (4.26)	-	-	-
**Exciting personality**	-	0.93 ^a^	0.35 (NS)	0.08
wife	7.86 (3.33)	-	-	-
husband	8.15 (3.63)	-	-	-
**Creativity and artistry**		5.59 ^b^	< 0.0001	0.59
wife	9.35 (2.58)	-	-	-
husband	7.8 (2.9)	-	-	-
**house care**	-	10.62 ^b^	< 0.0001	1.65
wife	11.48 (2.02)	-	-	-
husband	7.3 (3.03)	-	-	-
**Intelligence**	-	3.74 ^b^	< 0.0001	0.34
wife	7.16 (2.67)	-	-	-
husband	6.24 (2.83)	-	-	-
**Good earning capacity**	-	11.57 ^a^	< 0.0001	2.1
wife	6.17 (2.55)	-	-	-
husband	11.44 (2.45)	-	-	-
**Wanting children**	-	1.56 ^b^	0.11 (NS)	0.15
wife	11.11 (2.13)	-	-	-
husband	10.75 (2.47)	-	-	-
**Being easygoing**		3.60 ^a^	< 0.0001	.39
wife	5.97 (2.76)	-	-	-
husband	7.14 (3.39)	-	-	-
**Good heredity**	-	2.40 ^a^	0.01	0.24
wife	4.56 (3.09)	-	-	-
husband	5.39 (3.74)	-	-	-
**College education**	-	4.31 ^a^	< 0.0001	0.36
wife	8.27 (3.21)	-	-	-
husband	9.4 (2.95)	-	-	-
**Physical attractiveness**	-	4.16 ^b^	< 0.0001	0.4
wife	8.43 (2.98)	-	-	-
husband	7.15 (3.37)	-	-	-
**Health status**	-	2.85 ^a^	0.004	0.32
wife	4.28 (2.92)	-	-	-
husband	5.32 (3.46)	-	-	-

^b^ Based on negative ranks (wife < husband)

^a^ Based on positive ranks (wife > husband)

**Table 3.** Gender Differences in Mate Preferences: Ranking Instrument

	Mean (SD)	z	P value	d
**Kindness and sympathy**	-	0.71 ^a^	0.47 (NS)	0.04
Wife	2.19 (1.58)	-	-	-
Husband	2.12 (1.64)	-	-	-
**Religiousness**	-	1.77 ^b^	0.07 (NS)	0.12
Wife	5.11(4.03)	-	-	-
Husband	5.63 (4.26)	-	-	-
**Exciting personality**	-	0.93 ^b^	0.35 (NS)	0.08
Wife	7.86 (3.33)	-	-	-
Husband	8.15 (3.63)	-	-	-
**Creativity and artistry**	-	5.59 ^a^	< 0.0001	0.59
Wife	9.35 (2.58)	-	-	-
Husband	7.8 (2.9)	-	-	-
**house care**	-	10.62 ^a^	< 0.0001	1.65
Wife	11.48 (2.02)	-	-	-
Husband	7.3 (3.03)	-	-	-
**Intelligence**	-	3.74 ^a^	< 0.0001	0.34
Wife	7.16 (2.67)	-	-	-
Husband	6.24 (2.83)	-	-	-

^a^ Based on positive ranks (wife > husband).

^b^ Based on negative ranks (wife < husband)

**Table 4.** Gender Differences in Mate Preferences: Ranking Instrument

	Mean (SD)	z	P value	d
**Good earning capacity**	-	11.57 ^a^	< 0.0001	2.1
Wife	6.17 (2.55)	-	-	-
Husband	11.44 (2.45)	-	-	-
**Wanting children**	-	1.56 ^b^	0.11 (NS)	0.15
Wife	11.11 (2.13)	-	-	-
Husband	10.75 (2.47)	-	-	-
**Being easygoing**	-	3.60 ^a^	< 0.0001	0.39
Wife	5.97 (2.76)	-	-	-
Husband	7.14 (3.39)	-	-	-
**Good heredity**	-	2.40 ^a^	0.01	0.24
Wife	4.56 (3.09)	-	-	-
Husband	5.39 (3.74)	-	-	-
**College education**	-	4.31 ^a^	< 0.0001	0.36
Wife	8.27 (3.21)	-	-	-
Husband	9.4 (2.95)	-	-	-
**Physical attractiveness**	-	4.16 ^b^	< 0.0001	0.4
Wife	8.43 (2.98)	-	-	-
Husband	7.15 (3.37)	-	-	-
**Health**	-	2.85 ^a^	0.004	0.32
Wife	4.28 (2.92)	-	-	-
Husband	5.32 (3.46)	-	-	-

^a^ Based on positive ranks (wife > husband)

^b^ Based on negative ranks (wife < husband)

The highest differences were related to good earning capacity, house care, and physical attractiveness. The least differences were in kindness and sympathy, exciting personality and wanting children. Results in Tables 5, 6, 7, 8, and 9 show the gender differences in most of mate preferences except 5 criteria in dating couples (chastity, ambition, similar political backgrounds, mutual attraction/love, intelligence). Gender differences in the good cook and good financial prospect items have large and medium effect sizes, respectively. The most important mate preferences from the view of wives were dependable character, desire for home and children, emotional stability, and maturity, respectively; from the view of husbands they were dependable character, desire for home and children, and mutual attraction/love, respectively. 

**Table 5.** Gender Differences in Mate Preferences

Mate Preference	Mean (SD)	Wilcoxon’s Gender Differences		
		Z	P Value	d
**Being a good cook (helping in cooking)**	-	6.57 ^a^	< 0.0001	1.59
wife	1.52 (0.72)	-	-	-
Husband	2.07 (0.76)	-	-	-
**House care**	-	5.54 ^a^	< 0.0001	0.61
Wife	1.95 (0.79)	-	-	-
Husband	2.41 (0.70)	-	-	-
**Pleasant disposition**	-	3.01 ^b^	0.003	0.31
Wife	2.90 (0.33)	-	-	-
Husband	2.76 (0.55)	-	-	-
**Sociability**	-	4.36 ^b^	< 0.0001	0.44
Wife	2.94 (0.24)	-	-	-
Husband	2.77 (0.52)	-	-	-
**Similar educational background**	-	2.79 ^b^	0.005	0.23
Wife	2 (0.86)	-	-	-
Husband	1.78 (0.85)	-	-	-
**Refinement, neatness**	-	2.81 ^b^	0.005	0.25
Wife	2.92 (0.29)	-	-	-
Husband	2.82 (0.49)	-	-	-
**Good financial prospect**	-	6.26 ^b^	< 0.0001	0.72
Wife	2.26 (0.66)	-	-	-
Husband	1.72 (0.85)	-	-	-
**Chastity (no previous sexual intercourse)**	-	1.91 ^a^	0.056	0.30
Wife	2.64 (0.72)	-	-	-
Husband	2.75 (0.60)	-	-	-
**Dependable character**	-	2.00 ^b^	0.04	0.33
Wife	3 (0)	-	-	-
Husband	2.98 (0.13)	-	-	-
**Emotional stability and maturity**	-	1.98 ^b^	0.04	0.23
Wife	2.96 (0.24)	-	-	-
Husband	2.89 (0.37)	-	-	-
**Desire for home and children**	-	2.52 ^b^	0.01	.31
Wife	2.98 (0.13)	-	-	-
Husband	2.92 (0.26)	-	-	-
**Favorable social status**	-	4.65 ^b^	< 0.0001	0.44
Wife	2.51 (0.60)	-	-	-
Husband	2.22 (0.70)	-	-	-
**Good appearance**		2.60 ^a^	0.009	0.23
Wife	2.25 (0.75)	-	-	-
Husband	2.42 (0.68)	-	-	-
**Similar religious background**	-	2.74 ^b^	0.006	0.25
Wife	2.70 (0.60)	-	-	-
Husband	2.53 (0.76)	-	-	-
**Ambition**	-	1.62 ^a^	0.10	0.15
Wife	1.28 (0.93)	-	-	-
Husband	1.42 (0.87)	-	-	-
**industriousness**	-	3.99 ^b^	< 0.0001	.40
Wife	2.77 (0.48)	-	-	-
Husband	2.55 (0.60)	-	-	-
**Similar political background**	-	1.04 ^b^	0.29	0.10
Wife	1.20 (1.08)	-	-	-
Husband	1.10 (0.90)	-	-	-
**Mutual attraction/love**	-	0.03 ^b^	0.97	0
Wife	2.92 (0.28)	-	-	-
Husband	2.92 (0.33)	-	-	-
**Good health status**	-	2.41 ^b^	0.01	0.24
Wife	2.94 (0.25)	-	-	-
Husband	2.87 (0.34)	-	-	-
**Education**	-	2.26 ^b^	0.02	0.2
Wife	2.25 (0.78)	-	-	-
Husband	2.09 (0.82)	-	-	-
**Intelligence**	-	0.63 ^b^	0.52	0.04
Wife	2.61 (0.59)	-	-	-
Husband	2.58 (0.63)	-	-	-
**Cultural similarity between families**	-	2.38 ^b^	0.01	0.24
Wife	2.75 (0.50)	-	-	-
Husband	2.61 (0.65)	-	-	-
**Financial similarity between families**	-	3.02 ^b^	0.002	0.31
Wife	2.23 (0.73)	-	-	-
Husband	1.98 (.88)	-	-	-

^a^ Based on positive ranks (wife > husband).

^b^ Based on negative ranks (wife < husband).

**Table 6.** Gender Differences in Mate Preferences

	Mean (SD)	z	P Value	d
**Being a good cook (helping in cooking)**	-	6.57 ^a^	< 0.0001	1.59
Wife	1.52 (0.72)	-	-	-
Husband	2.07 (0.76)	-	-	-
**House care**	-	5.54 ^a^	< 0.0001	0.61
Wife	1.95 (0.79)	-	-	-
Husband	2.41 (0.70)	-	-	-
**Pleasant disposition**	-	3.01 ^b^	0.003	0.31
Wife	2.90 (0.33)	-	-	-
Husband	2.76 (0.55)	-	-	-
**Sociability**	-	4.36 ^b^	< 0.0001	0.44
Wife	2.94 (0.24)	-	-	-
Husband	2.77 (0.52)	-	-	-
**Similar educational background**	-	2.79 ^b^	0.005	0.23
Wife	2 (0.86)	-	-	-
Husband	1.78 (0.85)	-	-	-
**Refinement, neatness**	-	2.81 ^b^	0.005	0.25
Wife	2.92 (0.29)	-	-	-
Husband	2.82 (0.49)	-	-	-

^a^ Based on positive ranks (wife > husband).

^b^ Based on negative ranks (wife < husband).

**Table 7.** Gender Differences in Mate Preferences

	Mean (SD)	z	P Value	d
**Good financial prospect**	-	6.26 ^a^	< 0.0001	0.72
Wife	2.26 (0.66)	-	-	-
Husband	1.72 (0.85)	-	-	-
**Chastity (no previous sexual intercourse)**	-	1.91 ^b^	0.056	0.3
Wife	2.64 (0.72)	-	-	-
Husband	2.75 (0.60)	-	-	-
**Dependable character**	-	2.00 ^a^	0.04	0.33
Wife	3 (0)	-	-	-
Husband	2.98 (0.13)	-	-	-
**Emotional stability and maturity**	-	1.98 ^a^	0.04	0.23
Wife	2.96 (.24)	-	-	-
Husband	2.89 (.37)	-	-	-
**Desire for home and children**	-	2.52 ^a^	0.01	0.31
Wife	2.98 (0.13)	-	-	-
Husband	2.92 (0.26)	-	-	-
**Favorable social status**	-	4.65 ^a^	< 0.0001	0.44
Wife	2.51 (0.60)	-	-	-
Husband	2.22 (0.70)	-	-	-

^a^ Based on Negative Ranks (wife < husband)

^b^ Based on Positive Ranks (wife > husband)

**Table 8.** Gender Differences in Mate Preferences

	Mean (SD)	z	P Value	d
**Good appearance**	-	2.60 ^a^	0.009	0.23
Wife	2.25 (0.75)	-	-	-
Husband	2.42 (0.68)	-	-	-
**Similar religious background**	-	2.74 ^b^	0.006	0.25
Wife	2.70 (0.60)	-	-	-
Husband	2.53 (0.76)	-	-	-
**Ambition**	-	1.62 ^a^	0.10	0.15
Wife	1.28 (0.93)	-	-	-
Husband	1.42 (0.87)	-	-	-
**Industriousness**	-	3.99 ^b^	< 0.0001	0.4
Wife	2.77 (0.48)	-	-	-
Husband	2.55 (0.60)	-	-	-
**Similar political background**	-	1.04 ^b^	0.29	0.1
Wife	1.20 (1.08)	-	-	-
Husband	1.10 (0.90)	-	-	-
**Mutual attraction/love**	-	0.03 ^b^	0.97	0
Wife	2.92 (0.28)	-	-	-
Husband	2.92 (0.33)	-	-	-

^a^ Based on Positive Ranks (wife > husband)

^b^ Based on Negative Ranks (wife < husband)

**Table 9.** Gender Differences in Mate Preferences

	Mean (SD)	z	P Value	d
**Good health status**	-	2.41 ^a^	0.01	0.24
Wife	2.94 (0.25)	-	-	-
Husband	2.87 (0.34)	-	-	-
**Education**	-	2.26 ^a^	0.02	2
Wife	2.25 (0.78)	-	-	-
Husband	2.09 (0.82)			
**Intelligence**	-	0.63 ^a^	0.52	0.04
Wife	2.61 (0.59)	-	-	-
Husband	2.58 (0.63)	-	-	-
**Cultural similarity between families**	-	2.38 ^a^	0.01	0.24
Wife	2.75 (0.50)	-	-	-
Husband	2.61 (0.65)	-	-	-
**Financial similarity between families**	-	3.02 ^a^	0.002	0.31
Wife	2.23 (0.73)	-	-	-
Husband	1.98 (0.88)	-	-	-

^a^ Based on Negative Ranks (wife < husband)

### 4.2. Qualitative Phase

Two themes were extracted from the data each of which included some categories and subcategories (Table 10). Due to the word count limitation, only some parts of the qualitative results (category or subcategory) which can explain the quantitative results better are describe in the discussion section. 

**Table 10.** Themes in Mate Selection From two Points of View

Category	Subcategory
**Gender Similarities in Mating Preferences**	
**Culture-religion**	Same culture
	Similar Religious believes
**Family**	Religious-belief-education, finance, ethics of family
	parentage
**Demographic**	Education
	Financial status
**Individual criteria**	appearance
	love
	Ethics and behavior
	Kindness and sympathy
	Couple communication
	sociability
	Financial opinion
	Getting affected by other people
**Opposite sex friendship (chastity)**	History of opposite sex friendship
**Health status**	addiction
**Politics**	Political idea
**Gender Differences in Mating Preferences**	
**Support**	Age difference
	Occupation (Financial capacity)
**Individual criteria**	Emotional stability and maturity
	Parenting capability
	Physical attractiveness
**Being easygoing**	veil
	Decision making
**Participation and empathy**	Participation in life
	Participation in sexual life
**Expectattion**	Cooking and house work
	Sexual expectancy

## 5. Discussion

Choosing a mate with all desired preferences is hard due to the limitation of available mates and little information about the important characteristics of potential mates (3, 7, 11); nonetheless, people try to choose the best. Comparison of the actual age differences and preferred age differences is one available method to check the validity of mate preferences. The actual age difference was 3.37 years (women younger than the men), while the preferred age difference was 3.93 years (women preferred their husband to be 4.01 years older, and men preferred their wives to be 3.88 years younger). These results demonstrate the validity of age difference criterion as a evaluation tool for mate preferences. Change et al. designed a study in 1982 to examine the persistent sex differences in the modern Chinese population. Their results showed the preferred age difference from the women’s view was 3.45 years older and from the men’s view 2.15 years younger. When they compared the results of 1982 with 2008, they noticed that the age difference between women and men has been constant during these years (16). Haghighizadeh et al. study on university students of Ahwaz - Iran, reported that 4 - 6 years was the appropriate age difference between wives and husbands (24). Cross-cultural study of Buss in 1989 showed that across 27 countries, the actual age difference was from 2.17 to 4.92 years (11). University students of Ahwaz preferred the marriage age of 26 - 30 years for boys and 20 - 25 years for girls (24). Harazi et al. in a survey on Shahid Sadooghi University students of Yazd-Iran, revealed that the preferred marriage age for boys and girls was 26.28 and 22.17 years, respectively (25). In Buss’s study, the average preferred marriage age of men was indicated as 27.5 years and for their mates 24.8 years .Women preferred 25.4 years for themselves and 28.8 years for their mates (11). All results of this study and others, support the evolution-based theory stating that men prefer and choose younger women for their high reproductivity capacity (11, 16, 17). Besides this theory, in a deep individual interview during the qualitative phase, our participants explained reasons other than the reproductivity capacity for preferring and choosing older husbands and younger wives. The aging phenomenon, starting earlier in women than men, was one important reason they mentioned. One male participant said “earlier puberty as well as pregnancy and menopause in women cause earlier aging; their sexual desire decreases, and they become impatient and boring. Well, when husband come home with enthusiasm and passion and needs sex, wife does not accept and this leads to quarrel”. Another man said “women prefer older men as a mate because they have larger penises compared with younger ones” and a female participant said “I do not like a younger man as a mate because he does not have enough experiences, sophistication and skills to manage and solve the problems. A man should support his wife and a women should be able to rely on her spouse”. Other male participant mentioned “Women prefer older men because they want to rely on them”. Thus, a younger woman and an older man as a couple can guarantee a reasonable and understanding life besides having better sex; hence, it is not only about the fertility. One research supportive of our qualitative data is Burrows’ study in 2013. Her Data suggested that the biological-reproductive theory of age hypergamy is incomplete and supports a cultural reproduction model of the gender role (26).

In the current study, some preferences were more important for men and some for women. “Good earning capacity”, “Good financial prospect” with large effect sizes received the highest rates and “College education”, “health status”, “Favorable social status”, and “Industriousness” with small effect sizes received a little more important rates from women. Men dedicated the more important rate to “Intelligence”, “Creativity” with small and medium effect sizes. Chang et al. reported; “College education”, “health status”, ”Favorable social status” and “Industriousness” as strong guarantees of income were important from the wives’ views (16). Sefcek believed that as a whole, woman asks herself: could he and will he invest for me during the common life (27) ? In an interview, women of two groups emphasized on not being stingy in a potential mate. One woman said “I would like a man as my future husband who can have independent resources and pay money to buy anything without niggardliness. You know some men have more money but do not spend it for their family. I hate stingy people especially stingy man in the family”. Men in the qualitative phase explained the reason of the importance of “Intelligence”, “Creativity” in a mother’s role in raising and taking care of children. One participant said “I would like to marry an intelligent and creative girl, because she has an important effect on raising our children”.

Based on the evolutionary theory, physical attractiveness is a prediction criterion of fertility (10, 11); but the results of this study do not confirm it. Significant gender differences occurred for “physical attractiveness”, but it was more important from the view of wives than husbands. Participants had different opinions about “physical attractiveness” and “good appearance”. Husbands gave a little more score to “good appearance”. In the qualitative phase, participants described that “physical attractiveness” is different from” good appearance”; someone may not be beautiful or have good appearance, but she/he be physically attractive. They believed that beauty is more necessary for women but “physical attractiveness” for men. They did not mention any relation between physical attractiveness or good appearance and fertility. They require these qualifications to proud of their spouse among family members and friends and receive their confirmation. Appearance is necessary for men because of the sexual attraction. Men said “I like girl with plump bodies and cute faces”, “it has always been in my mind to marry a girl with a lovely and pleasing appearance”, “I like a girl with a sexy body and lustrous hair for more pleasure in sex”, “I like a beautiful girl as my wife to get my cousins’ confirmations”. Women said “men should be attractive and masculine because we can rely on them as the man of our lives” , “man must have a masculine body, not thin or small, and neither beautiful nor ugly appearance, in a way that people don’t call them ugly”. In some studies, gender differences were seen in physical attractiveness, but more preferred by men than women (11, 13, 15, 16, 28). Buss, et al. believed that steady climb in physical attractiveness preference in both genders is related to the environment, TV, and movies (17). Price et al. concluded that attractiveness of males and females has a significant role in human mating. For example, less attractive females express stronger tendency for less attractive men (29). In Eagly and Wood’s study there were no gender differences in “physical attractiveness” because it exchange with other preferential mating in mate selection for other gains (30). Berg confirmed that attractiveness is an important signal for a potential mate (31). Most researchers concluded that physical attractiveness is an index of fertility in the evolutionary theory, but none of them asked its reason from the participants. Their conclusions are theoretical, but not realistic. This claim becomes stronger where the physical attractiveness matter is influenced by TV and movies, affecting the sexual pleasure, which is not related to fertility.

Gender differences were seen in “house care” and “being a good cook” items. Husbands mentioned these qualifications more than wives. In Iranian families, women prefer to be responsible for the house work and cooking besides working outdoors. Most of them believe that house work is feminine and their husbands should not do it. Also, they do not like hiring domestic helpers, because they can’t trust or pay them easily. Wives mentioned “sociability”, “emotional stability and maturity”, “pleasant disposition”, “being easygoing” more than husbands which is similar to the Change and Buss’s studies (16, 17). One woman said "I like a man with good social relationships, ethical consideration, smiling face, independent decision makings, no whimsicality, and respectfulness". A man said "I like my wife to be polite, calm, and sociable, but I don’t want her to talk and smile with other men" Although these preferences can help couples have less conflicts, there is no theory to describe why there are gender differences regarding these variables in some cultures and over time (12).

As we see, selecting a marriage partner is more of a culturally-defined process. The selection rules vary widely from society to society and are often complex. Arranged marriages have been common in Iran. In this marriage type, all family members have roles in the mate selection, for both men and women (1). In our study, we can see family members, friends, and neighbors’ roles in mating selection in the qualitative data. A woman participant said “to be confirmed by family members, friends, and neighbors, a man must be sociable, responsible, and calm, in addition to having pleasant disposition and friendly behavior”. Another man said “a sociable woman can better take care of children and besides, she can get the confirmation of all family and friends”. As Saroukhani et al. said, despite some changes in mate selection pattern of Iran in the recent years, family and friends’ roles still have strong effects on couples’ decision makings (19).

Both genders believed that virginity is essential for a potential mate. Farahani et al. mentioned that although having sexual contact before marriage is disapproved in Iran especially for girls, we can see some young people have premarital relationships (32). Since premarital sex is not generally accepted by young people and their families, virginity is an important qualification in mate selection for both genders. Buss believed that this gender similarity of idea regarding “virginity” is similar to 38% of cultures and against 62% of them (9). Busche et al. found the same results.

Both men and women reported higher tendency to enter a new relationship when they had been single for a longer period of time (33). These differences show that it is a culture-based item. In our individual interviews, both genders had strong emphasis on “chastity” for a potential mate. A woman said “I do not like my future husband with a history of friendship with the opposite sex. He must not even shake hand with his female cousins.” another woman said “having girlfriends in the past is not important in a potential mate, but eithout sexual contacts”. Men said “she must not have a history of friendship with the opposite sex”, “if she has history of friendship with opposite sex, it is probable for her to have extramarital relationships in the future”. We can see that, not only sexual contact, but also friendship with opposite sex as a negative point is very important for both genders in mate selection. Both genders ranked “kindness and sympathy” and more than that “mutual attraction/love” higher than “good earning capacity” and “physical attractiveness”. Universal desire of both genders in some preferences such as “kindness and sympathy” and “mutual attraction/love” shows that these items are not culturally based (9).

Both genders ranked “religiosity” in the forth position. Buss et al. showed that religiosity is cultural and time-based, because in some cultures it has the minimum importance (15). gender difference was seen in Buss et al. study (17) and in time difference was observed in Chang et al. study considering the importance of “religiosity” in mate selection (16). Not only this study, but others such as Harazi et al. concluded that in an Islamic country, religiosity is obviously important for both genders in mate selection (25).

Wide-ranging conclusions can be derived from this study. First, the actual and preferred age difference between wives and husbands is almost similar, demonstrating that men try to seek a younger mate with their preferences. Second, in spite of socio-cultural changes, especially the outdoor roles of women, husbands preferr a mate who does the housework as well as being a good cook and women prefer a mate with “Good earning capacity”, “Good financial prospect” “College education”, “Favorable social status” and “Industriousness”. These results show the effect of gender-based growth of a person in Iranian families. Third, an interesting result which was different from other studies was the different meanings of “good appearance”, “beauty” and “physical attractiveness” from the view of these couples; they believed that “beauty” is more necessary for women but “physical attractiveness” for men. They did not mention any connection between “physical attractiveness” or “good appearance” and fertility. They required these qualifications to be proud of their spouse in front of family members and friends and get their confirmation. Good appearance is necessary because of sexual relationship.

The results confirm that we need a combined theoretical approach to comprehensively explain the view to the human mating process in addition to qualitative and quantitative studies to explore the real meaning of each preference from the view of young people, not just from the view of the existing theory; because probably we should add other new theories to this field.

### 5.1. Limitations and Strengths of Study

Our study was limited by its culturally-homogeneous sample in only one city of Iran, thus the generalizability of the ﬁndings to a more racially and ethnically diverse Iranian population is uncertain. Replication of this study in other diverse and larger samples would help us to confirm the results. Despite this limitation, the current study is unique to explore the real meaning of each preference in mates from the view of young people, not just from the view of the existing theory.

## References

[1] O'Neil D (2010). sex and marriage :Overview: Part 1 & 2. ..

[2] Breschi Marco, Manfredini Matteo, Mazzoni Stanislao (2010). Health and socio-demographic conditions as determinants of marriage and social mobility: Male partner choice in Sardinia, late 19th-early 20th centuries.. Demographic Research..

[3] Buss DavidM (1985). Human mate selection: Opposites are sometimes said to attract, but in fact we are likely to marry someone who is similar to us in almost every variable.. American Scientist..

[4] Manfredini M, Breschi M, Mazzoni S (2010). Spouse selection by health status and physical traits. Sardinia, 1856-1925.. Am J Phys Anthropol..

[5] Buss DavidM, Angleitner Alois (1989). Mate selection preferences in Germany and the United States.. Personal Individual Diff..

[6] Maatta Kaarina, Uusiautti Satu (2013). Who is the One? The Difficulty in Selecting the Partner.:.

[7] Buss DM, Schmitt DP (1993). Sexual strategies theory: an evolutionary perspective on human mating.. Psychol Rev..

[8] Abedi D, Farahbakhsh K (2001). A Comparison Betwwen Viwes of Male and Female Students In Choosing Spouse.. Teb Va Tazkieh..

[9] Buss DavidM (2006). Strategies of human mating.. Psihologijske teme..

[10] Shoemake ElizabethG (2007). Human mate selection theory: An integrated evolutionary and social approach.. J Scie Psych..

[11] Buss DavidM (1989). Sex differences in human mate preferences: Evolutionary hypotheses tested in 37 cultures.. Behav Brain Scie..

[12] Buss DavidM, Abbott Max, Angleitner Alois, Asherian Armen, Biaggio Angela, Blanco-Villasenor Angel (1990). International Preferences in Selecting Mates A Study of 37 Cultures.. J Cross-cultural Psych..

[13] Li NormanP, Valentine KatherineA, Patel Lily (2011). Mate preferences in the US and Singapore: A cross-cultural test of the mate preference priority model.. Personality Individual Diff..

[14] Mather RobertD (2006). Using evolutionary psychology to account for sex differences and similarities in psychological tendencies.. J Scie Psych..

[15] Buss DM (1988). The evolution of human intrasexual competition: tactics of mate attraction.. J Pers Soc Psychol..

[16] Chang Lei, Wang Yan, Shackelford ToddK, Buss DavidM (2011). Chinese mate preferences: Cultural evolution and continuity across a quarter of a century.. Personality Individual Diff..

[17] Buss DavidM, Shackelford ToddK, Kirkpatrick LeeA, Larsen RandyJ (2001). A half century of mate preferences: The cultural evolution of values.. J Marriage Family..

[18] Torabi Fatemeh, Baschieri Angela (2010). Ethnic differences in transition to first marriage in Iran: The role of marriage market, women's socio-economic status, and process of development.:.

[19] Saroukhani Bagher, Mogharabian Maryam (2011.). Modernist and Marriage, A Comparative Study Among Women Lived in Regions 1 And 11 of Tehran.. Journal of Culture-Communication Studies..

[20] Cohen J (1992). A power primer.. Psychol Bull..

[21] Cohen J (2013). Statistical Power Analysis for the Behavioral Sciences.:.

[22] Fritz CO, Morris PE, Richler JJ (2012). Effect size estimates: current use, calculations, and interpretation.. J Exp Psychol Gen..

[23] Graneheim UH, Lundman B (2004). Qualitative content analysis in nursing research: concepts, procedures and measures to achieve trustworthiness.. Nurse Educ Today..

[24] Haghighizadeh M, Karami Kh, Soltani T (2010.). The Criteria of Spouse Choosing in Viewpoint of Ahwaz Jundishapur University Of Medical Sciences Students.. J Health Scie..

[25] Harazi MA, Hossini Motlagh SM, Sadrian MR (2001). [Assessment The point of view of Shahid Sadooghi University students about marriage].. J Shahid Sadooghi Yazd Univ Med Scie..

[26] Burrows K (2013). Age preferences in dating advertisements by homosexuals and heterosexuals: from sociobiological to sociological explanations.. Arch Sex Behav..

[27] Sefcek JonA, Brumbach BarbaraH, Vasquez Geneva, Miller GeoffreyF (2007). The evolutionary psychology of human mate choice: How ecology, genes, fertility, and fashion influence mating strategies.. J Psych Human Sexual..

[28] Abbaszadeh M, Behi M, Darbandi MA, Yusefi M, Jamalzadeh F (2011). Critria of Spouse selection in the boy and girl students in Zabol University of Medical Sciences in 2008.. Quaterly J Rostamineh..

[29] Price ME, Pound N, Dunn J, Hopkins S, Kang J (2013). Body shape preferences: associations with rater body shape and sociosexuality.. PLoS One..

[30] Eagly AliceH, Wood Wendy (1999). The origins of sex differences in human behavior: Evolved dispositions versus social roles.. American Psychologist..

[31] Arantes J, Berg ME, Wearden JH (2013). Females' duration estimates of briefly-viewed male, but not female, photographs depend on attractiveness.. Evol Psychol..

[32] Farahani FK, Cleland J, Mehryar AH (2011). Associations between family factors and premarital heterosexual relationships among female college students in Tehran.. Int Perspect Sex Reprod Health..

[33] Busche L, Marks M, Oates K (2013). The effect of recent sexual activity on partner desirability:an evolutionary perspective.. J Social Evol Cultural Psych..

